# Digitale zorg op afstand in diabetesrichtlijnen

**DOI:** 10.1007/s12467-022-0640-2

**Published:** 2022-06-02

**Authors:** Nathalie Ekelemans

Digitale zorg op afstand heeft de afgelopen jaren een enorme vlucht genomen. Tijdens de coronapandemie hebben patiënten en zorgverleners in korte tijd veel ervaring opgedaan met de digitale tools die er nu al zijn. Zorg op afstand lijkt vooral voor de diabeteszorg aantrekkelijk, omdat diabetespatiënten veel digitaal meten en hun zorg voor een groot deel planbaar is. Technologie stelt de zorgverlener in staat om de zorg op een andere manier te organiseren en persoonsgerichter vorm te geven. Voor de meeste digitale toepassingen is er nog onvoldoende bewijs voor positieve effecten op de kwaliteit van zorg, de patiënttevredenheid of uitkomstmaten zoals tijd-en kostenbesparingen. Daardoor zijn er nog nauwelijks aanbevelingen opgenomen in de bestaande richtlijnen en (zorg)standaarden. De behoefte hieraan neemt wel toe. Daarom is recent een start gemaakt met het DiDia-project, *Digitale zorg op afstand in Diabetes richtlijnen*.

## Doel

Het NHG voert dit project uit in samenwerking met de Nederlandse Internisten Vereniging, het Kennisinstituut van de Federatie Medisch Specialisten, het National eHealth Living Lab, de Patiëntenfederatie Nederland, Diabetesvereniging Nederland en de Nederlandse Diabetes Federatie. ZonMw financiert het project.

Het doel is om concrete aanbevelingen te formuleren over effectieve, persoonsgerichte zorg op maat. Dit varieert wat betreft consult en contact voor volwassenen met diabetes mellitus type 1 en type 2. Hierbij worden recente ervaringen meegenomen van voor en tijdens de coronapandemie, waar mogelijk onderbouwd met beschikbaar wetenschappelijk onderzoek, ervaringen en geleerde lessen. De aanbevelingen worden opgenomen in de landelijke richtlijnen en (zorg)standaarden voor diabetes in de eerste en tweede lijn, in de informatie voor patiënten op Thuisarts.nl en in de NDF Toolkit persoonsgerichte diabeteszorg en preventie.

## Stand Van Zaken

Het gebruik van digitale middelen neemt zowel in de eerste als in de tweede lijn toe. Tijdens de coronapandemie is bij zorgverleners met name het gebruik van e-consulten (82% bij huisartsen), beeldbellen (55% bij huisartsen) en patiëntportalen (55% bij huisartsen) gestegen (E-healthmonitor, 2021). Een versnelling in de digitale zorgverlening doet zich ook voor in de tweede lijn, versterkt door continue glucosemetingen. Het is een belangrijke ontwikkeling dat patiënten en zorgverleners via bepaalde applicaties kunnen communiceren en meetwaarden direct, realtime kunnen doorgeven.

Prof. dr. Jako Burgers, huisarts en leerstoelhouder van het Nederlands Huisartsen Genootschap: ‘Heel belangrijk is het dat we de verschillende mogelijkheden goed weergeven in de richtlijnen, met betrouwbare en onafhankelijke informatie over de voor- en de nadelen. De huidige zorg is kwalitatief goed en mag dan ook zeker niet minder worden door zorg op afstand. Voor sommige patiënten-groepen kunnen we dankzij de digitale hulpmiddelen betere en toegankelijkere zorg bieden. Voor hen is “zorg op afstand” eigenlijk ook “zorg dichtbij”, omdat zij minder frequent naar het ziekenhuis hoeven. Dit geldt niet in alle gevallen. We willen dan ook systematisch informatie en ervaringen ophalen en onderbouwde keuzes maken. Neem het videoconsult, ofwel beeldbellen. Dat is veel toegepast tijdens de coronapandemie, maar er is steeds meer *evidence* dat dit wisselend wordt ervaren. De e-healthtoe-passingen moeten dan ook aansluiten bij de wensen van patiënten en zorgverleners, en de zorg zodanig onder-steunen dat deze van meerwaarde is voor de patiënt én de zorgverlener.’

## Knelpunten

Naast de positieve ervaringen zijn er ook knelpunten. Digitale opslag van metingen en gegevens biedt de mogelijkheid om deze direct te koppelen aan het elektronisch patiëntendossier en/of aan een persoonlijke gezondheids-omgeving (PGO), waardoor telemonitoring mogelijk wordt. Lastig hierbij is de grote variatie in het gebruik van digitale middelen – zowel bij patiënten als bij zorgverleners – en dat de data die worden ingewonnen via allerlei verschillende platforms toegankelijk zijn. Een groot knelpunt is dat de data hierdoor niet eenvoudig ingevoerd kunnen worden in het HIS. Die platforms zijn veelal gerelateerd aan de producenten van diabeteshulpmiddelen, zoals pomp en sensor. Het proces om tot heldere aanbevelingen te komen, vertraagt hierdoor. Daarnaast voldoen de meeste PGO’s nog niet aan de MedMij-criteria. Bovendien staat de commerciële markt van software-aan-bieders uniformiteit en samenwerking in de weg.

Burgers: ‘Juist de kanttekeningen willen we ook goed uitzoeken om gericht advies te geven wanneer iets wel bijdraagt aan goede zorg en wanneer niet. Bij het beoordelen van de digitale toepassingen kijken we naar verschillende aspecten: hoe werkt het, hoe betrouwbaar is het, hoe groot is de groep die er voordeel bij kan hebben, hoe staan zorgverleners en patiënten ertegenover, wat zijn de voorwaarden en hoe bed je het gebruik op een goede manier in binnen de aan te bieden zorg? Er zijn al veel mogelijkheden en de markt breidt zich snel uit. Dat kan ook meer ruis geven. We willen duidelijke randvoor-waarden formuleren; binnen de toepassingen die daaraan voldoen, is er keuzevrijheid.’

## Plan Van Aanpak

Om tot gedragen aanbevelingen te komen, is een plan van aanpak opgesteld, dat bestaat uit vijf fases. In de *eerste fase* worden ervaringen met zorg op afstand en implementatie-vraagstukken over zorg op afstand geïnventariseerd in focusgroepen met patiënten en zorgprofessionals uit de eerste en tweede lijn.

- Focusgroepen 1 en 2 bestaan uit mensen met type 1-diabetes en type 2-diabetes uit de eerste (10 patiënten met type 1-diabetes) en de tweede lijn (8 patiënten met type 1-diabetes en 2 patiënten met type 2-diabetes).
- Focusgroep 3 bestaat uit zorgverleners uit de eerste lijn: 4 huisartsen, 2 kader-huisartsen en 2-3 praktijkonder-steuners en 2 diëtisten.
- Focusgroep 4 bestaat uit zorgverleners uit de tweede lijn: 4 internisten, 2 kader-huisartsen, 2-3 diabetesver-pleegkundigen en 2 diëtisten.
- Focusgroep 5 bestaat uit overige zorgverleners: 2 podo-therapeuten, 2 apothekers, 2 fysiotherapeuten, 2 kader-huisartsen en 2 praktijkondersteuners.

Daarnaast worden kwantitatieve data verzameld, literatuuronderzoek gedaan en wordt een knelpunteninventarisatie verricht.

In de *tweede fase* worden door middel van een *invitational conference* ervaringen, knelpunten en bevorderende en belemmerende factoren bij de toepassing van zorg op afstand besproken en geprioriteerd om gezamenlijk tot consensus te komen welke knelpunten in de volgende fase worden meegenomen naar de richtlijnen.

De opgedane kennis, inzichten en ervaringen worden in de *derde fase* gebruikt om aanbevelingen te formuleren. Burgers: ‘De bestaande werkgroepen van de NHG-Standaard Diabetes mellitus type 2 en de diabetesrichtlijnen van de Nederlandse Internisten Vereniging (NIV) zullen volgens de gebruikelijke methode van richtlijnontwikkeling de uitgangsvragen uitwerken. Zij kijken kritisch naar wat uit de literatuur en uit de ervaringen komt.’

## Te Verwachten Resultaten

De praktijkervaringen, kwantitatieve data, het literatuur-onderzoek en de knelpunten leiden na de *invitational conference* tot een geprioriteerde lijst met aandachtspunten. Deze worden gebruikt bij het opstellen van specifieke aanbevelingen over zorg op afstand in de diabetesrichtlijnen van de eerste en de tweede lijn. Daarnaast leiden deze aandachtspunten tot een overzicht van bevorderende en belemmerende factoren die de basis vormen van een implementatieplan (*fase 4*), inclusief (rand)voorwaarden waaraan zorg op afstand zou moeten voldoen voor toepassing in de praktijk. In *fase 5* wordt het project afgerond met een eindrapportage.

## NDF Programma Hybride Diabeteszorg

Het DiDia-project is begin 2022 gestart en heeft een looptijd van achttien maanden. De NDF is zoals aangegeven één van de partners in het DiDia-traject. Corrine Brinkman, strategisch beleidsadviseur bij de NDF: ‘Binnen het NDF Programma *Hybride Diabeteszorg* zetten wij ons in voor de kwaliteitsslag die nodig is om ervaringen te “verzilveren” die gerelateerd zijn aan de opkomst van *smart diabetes devices* en die zijn opgedaan tijdens de coronapandemie. Denk bijvoorbeeld aan alternatieve consult-vormen. Daarbij is het idee dat effectieve, persoonsgerichte zorg op maat kan variëren wat betreft consult en type contact. Er zijn nog veel vragen die beantwoord moeten worden om kwaliteit te definiëren en te borgen. Dat kan goed in richtlijnen en we zijn daarom blij hier via DiDia een bijdrage aan te kunnen leveren. We zullen de opbrengsten van het traject opnemen in de NDF Zorgstandaard Diabetes en doorvertalen naar producten voor de NDF Toolkit persoonsgerichte diabeteszorg en preventie.’

## Persoonsgerichte Zorg

Burgers: ‘Ik vind het bijzonder waardevol dat we dit project met zo veel partijen uitvoeren. De coronapandemie heeft naast ellende ook positieve dingen gebracht. De onderlinge samenwerking is verbeterd, tussen zorgprofessionals onderling en tussen zorgprofessionals en patiënten. Dat moeten we zeker vasthouden. Ik ben heel enthousiast om specifiek op dit onderwerp, door het inzetten van digitale hulpmiddelen, het aanbod van zorg te vergroten en aan te passen aan de mogelijkheden van deze tijd en daardoor de zorg te verbeteren. Ik zou zorgverleners willen meegeven: wees niet bang om nieuwe digitale toepassingen uit te proberen. Het is niet zo dat de diabeteszorg straks volledig algoritmegestuurd wordt. Persoonsgerichte zorg blijft leidend. De toepassingen zijn er niet ter vervanging, maar ter ondersteuning van besluitvorming.’

